# Clinical and functioning outcomes during the establishment phase of Ukraine's community mental health teams: a descriptive analysis

**DOI:** 10.1016/j.lanepe.2025.101446

**Published:** 2025-09-03

**Authors:** Alisa Ladyk-Bryzghalova, Charles Zemp, Marjolaine Rivest-Beauregard, Oleksii Kostiuchenkov, Alison Schafer, Ben Adams, Dan Chisholm, Philip Hyland, Mel Ó Súird, Katerina Drakos, Iryna Mykychak, Jarno Habicht, Frédérique Vallières

**Affiliations:** a WHO Country Office in Ukraine, Kyiv, Ukraine; b Trinity Centre for Global Health, School of Psychology, Trinity College Dublin, Dublin, Ireland; c Department of Psychiatry, McGill University, Montréal, Canada; d Douglas Research Centre, Douglas Mental Health University Institute, Montréal, Canada; e Department of Mental Health, Brain Health, and Substance Use, WHO, Geneva, Switzerland; f Department of Psychology, Maynooth University, Maynooth, Ireland; g Coordination Center for Mental Health under the Cabinet of Ministers of Ukraine, Kyiv, Ukraine

**Keywords:** Ukraine, Community mental health teams, Mental healthcare reform, Severe mental illness, Clinical recovery

## Abstract

**Background:**

Ukraine's nationwide Community Mental Health Teams (CMHTs) programme is key to Ukraine's ongoing mental healthcare reform. No studies to date, however, have reported on the impact of Ukrainian CMHTs on service user clinical recovery. This study has two aims: (i) describe *who* the Ukrainian CMHTs are enrolling, *which* services they most provide, and *where* they are provided and (ii) identify whether any clinical and/or functional improvements were detectable among service users after six CMHT visits (intake + five follow-up visits) and, if so, identify principal predictors of such improvements.

**Methods:**

947 CMHT service users enrolled between April–December 2021 were assessed on clinical outcomes using the Clinical Global Improvement scale (CGI) and functional outcomes using WHO's Disability Assessment Schedule (WHODAS 2·0). Chi-square and Wilcoxon signed-rank tests were used to assess changes in CGI and WHODAS scores, respectively, at the fifth (or fourth) follow-up CMHT visit. Hierarchical multinomial logistic regression and hierarchical multiple linear regression identified predictors of clinical and functional improvement, respectively.

**Findings:**

Most service users were male, unemployed, and diagnosed with schizophrenia spectrum disorders. Among service users with available outcome data at both CMHT intake and the fifth (or fourth) follow-up visit, a significant decrease in disability scores was observed (Median_intake_ = 62·50, Median_follow-up_ = 58·33, *z* = −6·27, *p* < 0·001) and most service users' illness severity stabilised (n = 451/742, 60·8%) or improved (n = 243/742, 32·6%). Clinical stabilisation (compared to worsening) was predicted by being male and living <20 km from the CMHT office, while improvement was predicted by frequent receipt of pharmacological support and receiving CMHT care in non-conflict-exposed regions. Functional improvement was predicted by living between 20 and 100 km from the CMHT office, having a somatic comorbidity, more frequent receipt of psychosocial services for the service user's family, and more support for community integration.

**Interpretation:**

We found positive results associated with enrolment in Ukraine's CMHTs. Recommendations for future research and improvements to the CMHT programming are provided.

**Funding:**

Funded as part of the World Health Organization's Special Initiative for Mental Health.

**Research in context**

**Evidence before this study**

Recent global mental health policy calls for mental healthcare systems to be re-oriented away from the historically hegemonial biomedical paradigm—favouring the delivery of care in institutions or other inpatient facilities—towards community-based approaches of service delivery. In alignment with this, over the past decade, the government of Ukraine—with support from World Health Organization—has been gradually shifting their national mental healthcare model away from institutionalisation toward more community-based, recovery-oriented, and person-centred practices. A core component of this reform was the nationwide rollout of community mental health teams (CMHTs). Designed to provide comprehensive support to Ukrainians living with severe mental illness (SMI), a recent Commission convened by *The Lancet Psychiatry (‘The Commission’)* identified the scale-up of these CMHTs as an essential next step in Ukraine's ongoing reform process—further stipulating that any scale-up efforts must be evidence-informed.

In August 2023, we conducted a literature search of Google Scholar to identify studies published in English, up to 2022, that explored the effectiveness of CMHTs, as a specific form of community-based mental healthcare, to support the recovery of individuals living with SMI, both in general and specific to the Ukrainian context—where the mental health impacts of the ongoing war with the Russian Federation have been well-documented. Search terms included: mental health or mental wellbeing; severe mental illness; schizophrenia or depression or anxiety or bipolar; recovery or clinical recovery or functional recovery or rehabilitation; effective or effect or effectiveness; community or community-based mental healthcare; community mental health teams or mobile community mental health teams; Ukraine or Ukrainian or Eastern Europe or Eastern European. We updated this search in October 2024. While we found a wealth of literature speaking to the importance of community-based mental healthcare, there was minimal research that specifically focused on CMHTs. Moreover, and corroborating what *The Commission* highlights, we found some literature that details the deployment and utility of CMHTs within the Ukrainian context (primarily grey literature, which has not undergone peer review), but none evaluated the effectiveness of those CMHTs in supporting the recovery of enrolled service users.

**Added value of this study**

We conducted the first comprehensive analysis of Ukraine's CMHT service model. We evaluated how well the CMHTs are meeting key programmatic performance indicators, and identified their effectiveness in improving important clinical outcomes among a sample of N = 947 Ukrainians diagnosed with a SMI who were enrolled during the ‘service establishment’ phase of Ukraine's CMHT service model. We found support that the Ukrainian CMHT service model is in alignment with a biopsychosocial approach to mental health care, effectively delivering care to individuals *within their community*. We also found that most individuals enrolled in the CMHT programme for a minimum of six CMHT visits over an average of 9·9 weeks (SD = 4·9), demonstrated stabilisation or improvement in illness symptom severity and had significantly lower levels of functional impairment. Furthermore, we identified both service user and service-related factors that were found to predict these outcomes. Through contextualising our findings, we recommend ways the Ukrainian CMHT service model can be improved to better support the recovery process for Ukrainians living with SMI.

**Implications of all the available evidence**

In direct response to recommendations from *The Commission*, our study provides much needed initial evidence around a central component of Ukraine's ongoing mental healthcare reform process. Demonstrating the Ukrainian CMHTs' effectiveness in improving clinical outcomes, identifying predictors of these improvements, and reflecting on successes and gaps in service provision, this study provides important avenues for future research, as well as actionable insights that can inform the country's recommended scale-up of CMHT programming.

## Introduction

Known for its high institutionalisation rates,^1^ Ukraine's mental health system is characterised by an extensive network of psychiatric services, which, collectively, account for 86·7% of Ukraine's mental health budget.^2^ In line with recent trends in global mental health policy and international governance standards,^1^ Ukraine's first concept note on mental healthcare, approved in December 2017, paved the way for deinstitutionalisation through the initiation of a community-based service delivery model that promotes community reintegration as a way to prevent further admissions.^3^ Supported by the World Health Organisation (WHO), under its Special Initiative for Mental Health (WHO-SIMH), the Ministry of Health Ukraine (MoH; Міністерство охорони здоров'я Украïни) launched their nationwide Community Mental Health Teams (CMHTs) programme in 2021 as a course of broader health care reform.^4^

Historically, this nationwide roll-out of CMHTs was preceded by two consecutive pilots in 2016 and 2020.^3^^,^^5^ The 2016 pilot deployed CMHTs in four psychiatric hospitals as part of the WHO's emergency programming targeting individuals with severe mental illness (SMI), including those displaced from conflict-affected communities. This initial service model, developed with the Ukrainian Research Institute of Social and Forensic Psychiatry and Drug Abuse, was well accepted by service users and staff, many with inpatient psychiatry experience. However, stigma, limited awareness of community-based care and therefore low cooperation, regulatory gaps, and regional needs highlighted the need for further adaptation.^5^

The second pilot in 2020—initiated amid a new phase of healthcare reform particularly focused on specialised services and the approval of Ukraine's first-ever national Mental Health Action Plan in 2021^6^—introduced 15 additional CMHTs, distributing them evenly across the country.^3^ WHO ensured training and supervision for these CMHTs' staff, covered operational costs, and provided ongoing technical guidance to health service managers, in consultation with the MoH. As in 2016, services were embedded into inpatient psychiatric facilities, recruiting those with SMI following discharge from hospitals. This second pilot, however, further included service specifications, standard operating procedures (SoPs) and key performance indicators (Table 1), along with a training programme for CMHT staff.^3^ The learnings of both pilots were thus central to informing the MoH's nationwide rollout directive of June 2021.^7^ Under this roll-out, the CMHT programme expanded to 87 CMHTs across 23 oblasts (WHO, unpublished), prior to suspending operations in certain oblasts following the escalation of the war in 2022.

**Table 1:** Key indicators for monitoring and evaluating performance of a Ukrainian CMHT.

Indicator	Target for one team
Duration of treatment	≥6 months
Average number of visits to one service user per month	4 visits
Ratio of psychosocial and pharmacological interventions^a^	psychosocial ≥ 50%, pharmacological ≤ 49%
Ratio of community-based and office-based visits^b^	community-based ≥ 50%, office-based ≤ 49%

a Psychosocial interventions reference only those provided to the service user themselves (i.e., not including psychosocial services for the service user's family).

b Visits are referred to as meetings with service user at their home, in another place in their community (e.g., park), in the office of the CMHT, at another health facility (e.g., primary health care clinic), online, and via phone consultations.

Today, CMHTs in Ukraine include a psychiatrist, psychologist, social worker, and nurse jointly responsible for conducting assessments, regular follow-up visits, facilitating recovery-oriented and person-centred care planning, providing a set of pharmacological and psychosocial interventions, engaging other services available in the community (e.g., support for accessing education, housing, employment, social protection services, leisure activities and peer support), and supporting family doctor registration, regular physical examinations and treatment for physical health conditions.^3^

A recent report by ‘*The Lancet Psychiatry* Commission on mental health in Ukraine’ (hereafter referred to as ‘*The Commission*’^1^) recommended the expansion of the existing CMHT programme as part of Ukraine's continued mental healthcare reform process. However, *The Commission* also cites that limited research on global CMHT programming to date has resulted in an “acute need for applied health research” to ensure that its recommended scale-up is evidence-based.^1^^(p922)^ This gap is particularly important given the absence of research on CMHTs in Ukraine, where, among other cultural factors, significant levels of mental health stigma and distrust of psychiatric services prevail.^8^ Moreover, ascertaining the effectiveness of and, indeed, recommending any improvements to CMHT programmes is especially pertinent given the documented mental health impacts of the war in Ukraine, both among the Ukrainian population and to the mental health infrastructure.^1^

Therefore, and aligned with the call from *The Commission*,^1^ this study offers findings from the nationwide rollout stage—or the ‘service establishment’ phase–of Ukraine's CMHT programme. Specifically, we offer a comprehensive description of *who* was enrolled during the ‘service establishment’ phase of CMHT programming in Ukraine, *which* services were most provided, and *where* they were provided. As a second objective, we sought to identify whether any clinical and/or functional improvements were detectable after six CMHT visits (intake + five follow-up visits) and, if so, identify the principal predictors of such improvements.

## Methods

### Participants and procedures

**Fig. 1:**
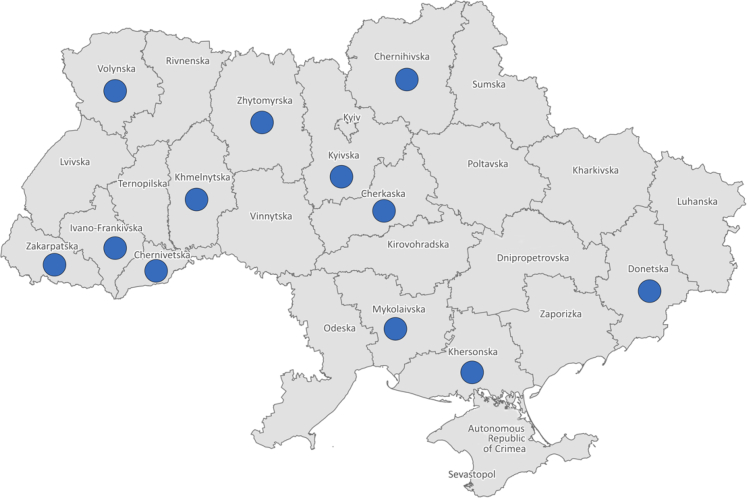
**Map of Ukraine demonstrating the oblasts (n = 12) where CMHTs were deployed with support of WHO in 2021**.

We undertook a secondary analysis of data routinely collected from *all* (N = 947) service users enrolled by 14 CMHTs receiving direct support from and agreeing to share service user data with WHO during the ‘service establishment’ phase (5 April–31 December 2021). These 14 CMHTs were deployed in 14 distinct psychiatric facilities, across 12 *oblasts* (administrative regions) in Ukraine; representing broad geographic coverage with CMHTs operating in half of the country's oblasts (Fig. 1; three CMHTs were deployed in Donetska oblast, with direct exposure to conflict prior to 2022). A subset of this sample was then selected based on those who had received *at least* five follow-up CMHT visits, to identify whether any clinical and/or functional improvements were observable during the ‘service establishment’ phase. This number of visits was chosen to best reflect the balance between maximising the timeframe of duration of CMHT care and retaining the most data for analytical purposes. Please see Supplementary Table S1 for the number of service users with data at CMHT intake and the first 10 follow-up visits, corresponding to the ‘service establishment’ phase.

### Variables and measures

#### Sociodemographic variables

Sociodemographic variables included: sex, age, distance from the CMHT office (in km), employment status, education level, and location (i.e., oblast). Both ‘distance from the CMHT office (in km)’ and ‘education level’, originally recorded categorically (Table 2), were dichotomised into ‘living <20 km from CMHT’ (=0) and ‘living between 20 and 100 km from CMHT’ (=1) and ‘has not completed tertiary education’ (=0) and ‘completed tertiary education’ (=1). Similarly, employment status was grouped into six categories (Table 2) and recoded into three categories–‘employed’ (=1), ‘unemployed’ (=2), and ‘other occupations’ (=3).

**Table 2:** Sociodemographic and clinical characteristics of all (N = 947) service users enrolled by 14 CMHTs during 5 April–31 December 2021.

	n^a^	%^a^	Mean (SD, range)
**Sociodemographic characteristics**			
**Sex**			
Male	504	53·4	–
Female	440	46·6	–
**Age**	935	–	44·2 (14·1, 18–93)
Male			41·2 (12·9, 18–93)
Female			47·7 (14·6, 18–92)
**Distance that CMHTs drive to reach services users in the community**			
<5 km	279	30·0	–
<20 km	521	56·1	–
<50 km	95	10·2	–
<100 km	34	3·7	–
**Employment status**			
Employed (full-time)	50	5·4	–
Employed (part-time)	58	6·2	–
Self-employed	20	2·1	–
Unemployed (but registered at the employment centre)	7	0·8	–
Unemployed (and not registered at the employment centre)	675	72·4	–
Other occupation^b^	122	13·1	–
**Education**			
Primary or incomplete secondary education	47	5·0	–
Basic secondary education	182	19·5	–
Vocational-technical secondary education	432	46·3	–
Incomplete tertiary education	64	6·9	–
Tertiary education	207	22·2	–
**Location**			
Zhytomyrska	54	5·7	–
Khersonska	96	10·1	–
Zakarpatska	43	4·5	–
Chernivetska	89	9·4	–
Kyivska	48	5·1	–
Khmelnytska	72	7·6	–
Donetska	234	24·7	–
Mykolaivska	76	8·0	–
Cherkaska	42	4·4	–
Volynska	72	7·6	–
Chernihivska	75	7·9	–
Ivano-Frankivska	46	4·9	–

	**n**^a^	**%**	**Mean (SD)**

**Clinical characteristics**			
**Referral source**			
Outpatient psychiatrist	441	47·0	–
Self-referral	232	24·7	–
Inpatient psychiatrist	178	19·0	–
Family doctor	35	3·7	–
Social service	1	0·1	–
Other^c^	51	5·4	–
**CGI Intake score (CGI-S)**	936	–	5·00 (1·00)
**No. of psychiatric hospitalisations in 12 months prior to intake**	930	–	0·89 (0·98)
0	391	42·0	–
1	311	33·4	–
2	185	19·9	–
3+	43	4·5	
**WHODAS summary score at intake**	888	–	61·15 (18·79)
**WHODAS summary score at fifth (or fourth) follow-up visit**	410	–	57·39 (16·85)
**Change in WHODAS score**	304	–	−6·68 (19·38)
**ICD-10 diagnosis**			
Schizophrenia, schizotypal, and delusional disorders (F20-29)	669	72·3	–
Mood [affective] disorders (F30-39)	109	11·8	–
Anxiety, dissociative, stress-related, somatoform, and other non-psychotic disorders (F40-48)	46	5·0	–
Other (F0–09; 50–59; 60–69; 70–79)	101	10·9	–
**Presence of somatic comorbidity**			
Yes	307	32·5	–
No	637	67·5	–

SD = standard deviation.

a Not including missing data, where applicable.

b Other occupation categories included: manages household, pensioner and manages household, on parental leave, studying, pensioner.

c Other referral sources can include homeless shelters, a relative, a friend, etc.

#### Clinical variables

##### Clinical improvement

CMHTs monitored clinical improvement using the Clinical Global Improvement (CGI) scale, which has previously been used in clinical research in Ukraine^9^ and is endorsed by the MoH for clinical use.^10^ Chosen for its brevity, practicality, and ease of administration in clinical settings,^11^ the CGI demonstrates good concurrent validity with other measures of psychiatric indications.^11^ The CGI comprises two single-item measures: (i) CGI-S (Severity) and (ii) CGI-I (Improvement). Both measures were rated by a CMHT member using a seven-point Likert scale. CGI-S assesses symptom severity at intake, with scores ranging from 1 = ‘normal’ to 7 = ‘among the most extremely ill patients’, reflecting initial severity. CGI-I scores range from 1 = ‘very much improved’ to 7 = ‘very much worse’, with a score of 4 corresponding to ‘no change’ (i.e., stabilisation), relative to symptom severity at intake. Within Ukraine's CMHT programme, the CGI-I is meant to be collected at every follow-up visit.

##### Functional improvement

CMHTs monitored functional improvement using the 12-item version of the WHO Disability Assessment Schedule (WHODAS 2·0). The WHODAS 2·0 has demonstrated strong cross-cultural reliability and validity across international settings,^12^ and the 36-item version was previously validated in Ukraine.^13^ The 12-item WHODAS 2·0 was independently translated by two mental health professionals, and expert consensus on the translated version was gathered and incorporated before CMHT staff were trained on its use. Items capture the level of functioning over the last 30 days, with two items capturing each of the following six domains: cognition, mobility, self-care, getting along, life activities, and participation. Each item is scored according to how much difficulty the participant experienced executing respective actions (e.g., ‘taking care of your household responsibilities’, ‘washing your whole body’, ‘maintaining a friendship’) on a scale ranging from 0 (no difficulty) to 4 (extreme difficulty or cannot do). A summary score, weighing items based on level of severity, was calculated using the WHODAS complex scoring template, where higher scores suggest greater disability. The WHODAS 2·0 summary score is collected by CMHTs at intake and once a month thereafter.

##### ICD-10 diagnosis

ICD-10 diagnosis was recorded at intake per the 10th revision of the International Classification of Diseases (ICD-10). Diagnoses were recategorised into four categories: schizophrenia, schizotypal, and delusional disorders (F20-29 codes); mood (affective) disorders (F30-39 codes); anxiety, dissociative, stress-related, somatoform, and other non-psychotic disorders (F40-48 codes); and other disorders (e.g., personality disorders, dementia, and intellectual disability; Table 2). For regression analyses, diagnoses were subsequently dichotomised: ICD-10 F20 codes (=1) and all other ICD-10 codes (=0), due to low cell counts in the ICD-10 F40–48 category.

##### Somatic comorbidity

Somatic comorbidity was originally recorded categorically, with service users being assessed for a somatic disorder at intake, again using the ICD-10. For analytical purposes, somatic comorbidities were recoded dichotomously, yes (=1), or no (=0), for ‘presence of somatic comorbidity’.

##### Number of psychiatric hospitalisations in the 12 months prior to CMHT intake

The total number of psychiatric hospitalisations in the 12 months prior to CMHT intake was sourced by CMHTs from medical records at intake.

##### Referral source

The referral source to the CMHT was recorded during the CMHT intake visit. Possible referral sources included an outpatient psychiatrist, inpatient psychiatrist, family doctor, social service, self-referral, or ‘other’ (e.g., homeless shelters, a relative or friend, etc.).

##### Time since intake

Time since intake was calculated by storing visit dates as date variables in the format DD/MM/YYYY and calculating the time elapsed (in weeks) from the intake date to the fifth follow-up visit for each participant.

#### CMHT visit-related variables

##### Services provided

CMHTs could provide up to nine distinct services at each visit: pharmacological services, psychosocial services for the service user, psychosocial services for the service user's family, general health services, social services, connection with other services in the community (i.e., job centres, housing services, etc.), developing a recovery plan, developing a treatment plan, and services to support the individual's reintegration into their community (i.e., re-establishing social connections, findings new friends, etc.). For our analyses, we calculated the total number of times each service was administered to each service user across six visits (intake visit + five follow-up visits). Service users were assigned a score from 0 to 6 for each service, with ‘0’ corresponding to never having received that service across any of the six visits and ‘6’ corresponding to having received that service at each of the six visits.

##### Visit location

CMHT visits occur in one of six distinct locations: in the CMHT office, in another medical facility, online, via telephone, at the service user's home, or elsewhere in the community. Since a key performance indicator of CMHTs is to conduct most visits within the service user's community (Table 1), we created a ‘community outreach’ variable, which combined visits occurring ‘at the service user's home’ and ‘elsewhere in the community’. For analytic purposes, we calculated the total number of visits each service user had in each location (including ‘community outreach’) across six visits (intake visit + five follow-up visits), resulting in a score of 0–6 for each location. Higher scores thus indicate higher frequency of visits in that location.

### Data analysis

All statistical analyses were performed using SPSS (v29). Missing data were assessed using Little's MCAR (Chi-Square = 11·91, df = 5, *p* = 0·036), indicating that data were not missing completely at random. However, given the naturalistic nature of the data and the rolling intake of service users, there was no indication that missing data resulted from anything other than programme attrition or varying lengths of programme participation. Therefore, data analyses were performed using pairwise deletion, and differences between included and excluded cases were assessed using chi-square, ANOVA, and t-tests, as appropriate.

A chi-square test of independence was used to investigate clinical improvements (per the CGI-I) from intake to fifth follow-up visit. When CGI-I scores were missing at fifth follow-up, fourth follow-up visit scores were used. To account for non-normality, a Wilcoxon signed-rank test was used to assess changes in disability scores (per the WHODAS) over time. Since the WHODAS is only administered monthly, the fourth follow-up scores were used when fifth follow-up WHODAS scores were unavailable. This was considered acceptable, as the average duration between visits was only 1·98 weeks. Change in disability scores was calculated by subtracting the intake score from the score at fifth (or fourth) follow-up visit. This process generated a total disability change score, where a negative total change score corresponds to a reduction in disability and a positive total change score corresponds to an increase in disability.

Predictors of change in symptom severity (i.e., clinical improvement) and disability scores (i.e., functional improvement) at the fifth (or fourth) follow-up visit were identified using hierarchical multinomial logistic regression and hierarchical multiple linear regression analysis, respectively. The magnitude of associations for clinical improvement were reported as odds ratios (ORs). To generate the criterion variable for clinical improvement, CGI-I scores at fifth (or fourth) follow-up visit were re-categorised as ‘improved’ (=1–3 on the CGI-I), ‘stabilised’ (=4 on the CGI-I), and ‘worsening’ (=5–7 on the CGI-I). Two hierarchical multinomial logistic regressions were run, with both ‘stabilised’ and ‘worsening’ used as reference categories. This allowed for identification of unique predictors of clinical improvement over stabilisation (as opposed to improvement over worsening, which we considered to be crucial to better support CMHT service users who had already achieved sustained symptom stabilisation). Predictors in all hierarchical regressions were entered in the following order: sociodemographic variables (i.e., Step 1: age, sex, distance from the CMHT office, time since intake, employment status, education level, and location), clinical variables (i.e., Step 2: ICD-10 diagnosis, presence of somatic comorbidity, number of psychiatric hospitalisations in the 12 months prior to intake), and service-related variables (i.e., Step 3: average frequency of the nine possible services provided and the ratio of visits conducted as community outreach across all six visits). For regression analyses, location and community outreach were recoded into dichotomous variables. Location was recoded into oblasts that were conflict-exposed prior to 2022 (i.e., Donetska; =1) and oblasts that were not conflict-exposed prior to 2022 (i.e., all other oblasts; =0). Community outreach was recoded into service users who received CMHT care within their community for ≥50% of their six visits (=1) and service users who received CMHT care within their community for <50% of their six visits (=0), as per the programme's key performance indicators (Table 1).

### Ethics

Ethical approval was obtained from Trinity College Dublin on February 28, 2025 (Approval No. 3572) and from the Committee on Bioethics of Scientific Research (Комітет з біоетики наукових досліджень) of the Taras Shevchenko National University of Kyiv (Кнïвськнй Національний Університет імені Тараса Шевченка) on November 21, 2024 (Approval No. 7). At enrolment, all CMHT service users provided written consent for the collection and processing of their personal data—including for scientific purposes—in accordance with Ukrainian law. All service user data was anonymised prior to being transferred for analysis.

### Role of the funding source

The work of the Community Mental Health Teams (CMHTs), which is the focus of this study, was financially supported via WHO's Special Initiative for Mental Health (SIMH), receiving funding from the Swiss Agency for Development and Cooperation (SDC), the Norwegian Agency for Development Cooperation (NORAD), and the United States Agency for International Development (USAID). Funding from the NORAD support also contributed to a contract between WHO HQ and Trinity College Dublin, for the analysis of the CMHT data and draft write-up of the manuscript. Staff of the WHO on the authorship team contributed to the study conceptualisation and design, collection, analysis and interpretation of data, the writing of the paper, and the decision to submit the paper for publication.

## Results

### Objective 1

The average age of service users at intake was 44·2 years (SD = 14·1, Range = 18–93). The majority were male (n = 504, 53·4%), living within 20 km of the CMHT offices (n = 800, 86·1%), and unemployed (n = 682, 73·2%). The largest proportion had not completed tertiary education (n = 725, 77·7%) and were receiving CMHT care in the Donetska oblast (n = 234, 24·7%)—the only included oblast directly exposed to conflict. Most service users were diagnosed with schizophrenia, schizotypal or delusional disorders (ICD-10 F20–29 codes; n = 669, 72·3%) and did not present with a somatic comorbidity (n = 637, 67·5%). Service users were, on average, assessed as markedly ill on their initial CGI assessment (CGI-S; M = 5, SD = 1) and had moderately severe disability scores at intake (M = 61·15, SD = 18·79). Service users had been hospitalised an average of 0·89 times (SD = 0·98) in the previous 12 months, and the largest proportion was referred to the programme by an outpatient psychiatrist (n = 441, 47·0%). Table 2 summarises the sociodemographic and clinical characteristics of all enrolled service users.

The most frequently provided services were ‘psychosocial services (for service user)’, administered an average of 4·17 times (SD = 1·81) across all six visits, followed by pharmacological services (M = 2·73, SD = 10·71). The ratio of psychosocial services to pharmacological services provided was 2·06 (Median = 1·50, Coefficient of Dispersion = 0·82). Out of the six total visits included in the analysis, an average of 3·09 visits (SD = 1·91), were conducted as community outreach. The provision of specific services and location of CMHT visits are detailed further in Table 3.

**Table 3:** Proportion of CMHT services provided and visit location at intake and first five follow-up visits for all (N = 947) service users enrolled by 14 CMHTs during 5 April–31 December 2021.

	Intake	Follow-up visit 1	Follow-up visit 2	Follow-up visit 3	Follow-up visit 4	Follow-up visit 5	Total
	n (%)^a^						Mean (SD)
**Services provided**							
Pharmacological services	640 (67·7)	371 (41·0)	344 (39·9)	327 (39·9)	315 (40·3)	327 (43·7)	2·73 (1·71)
General health services	254 (26·9)	262 (29·0)	246 (28·5)	270 (33·0)	254 (32·5)	245 (32·8)	1·81 (1·60)
Psychosocial services (for service user)	730 (77·2)	628 (69·5)	611 (70·9)	558 (68·0)	537 (68·8)	508 (67·9)	4·17 (1·81)
Psychosocial services (for service user's family)	280 (29·6)	207 (22·9)	193 (22·4)	196 (23·9)	190 (24·3)	184 (24·6)	1·48 (1·50)
Social services	54 (5·7)	66 (7·3)	79 (9·2)	76 (9·3)	72 (9·2)	70 (9·4)	0·52 (0·87)
Connecting to other services	34 (3·6)	46 (5·1)	55 (6·4)	59 (7·2)	49 (6·3)	49 (6·6)	0·35 (0·81)
Support to integrate into the community	8 (0·8)	9 (1·0)	11 (1·3)	16 (1·9)	14 (1·8)	13 (1·8)	0·09 (0·39)
Developing a recovery plan	242 (25·7)	115 (12·7)	99 (11·5)	88 (10·7)	87 (11·2)	80 (10·7)	0·85 (1·09)
Developing a treatment plan	284 (30·2)	120 (13·3)	58 (6·7)	47 (5·7)	58 (7·4)	55 (7·4)	0·72 (0·80)
**Visit location**							
**Within institution**							
CMHT offices	435 (46·0)	310 (34·3)	245 (28·4)	235 (28·6)	187 (24·0)	167 (22·3)	1·35 (1·38)
Other medical institution	49 (5·2)	34 (3·8)	27 (3·1)	12 (1·5)	13 (1·7)	10 (1·3)	0·15 (0·57)
**Remote**							
Online	15 (1·6)	13 (1·4)	17 (2·0)	11 (1·3)	21 (2·7)	22 (2·9)	0·10 (0·45)
Telephone	12 (1·3)	44 (4·9)	67 (7·8)	61 (7·4)	60 (7·7)	66 (8·8)	0·33 (0·71)
**Community outreach**							3·09 (1·91)
Service user's home	413 (43·7)	429 (47·4)	411 (47·6)	407 (49·6)	414 (53·1)	397 (53·1)	2·61 (1·90)
Elsewhere in the community	21 (2·2)	75 (8·3)	96 (11·1)	95 (11·6)	85 (10·9)	86 (11·5)	0·48 (0·82)

SD = standard deviation.

a Not including missing data, where applicable.

### Objective 2

Of N = 947 service users enrolled during the CMHT ‘service establishment phase’, n = 748 (79·0%) received at least five follow-up CMHT visits. Of these 748 service users, 742 had CGI scores at intake and fifth (or fourth) follow-up visit, and were therefore included in the multinomial regression analysis. Of the 748 service users who received at least five follow-up visits, 304 had WHODAS scores at intake and fifth (or fourth) follow-up visit and were thus assessed for changes in functional improvement. Supplementary Tables S2 and S3, respectively, present the results of testing for differences across key variables between included service users and those with fewer than five follow-up visits (n = 199) and between those with (n = 304) and without (n = 444) WHODAS scores at fifth (or fourth) follow-up.

#### Clinical improvement and functional improvement after five (or four) follow-up visits

**Fig. 2:**
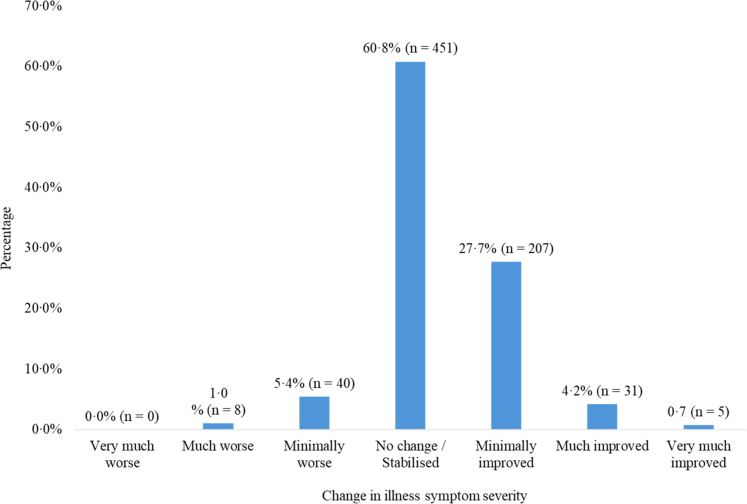
**Percentage of service users (n = 742) who at their fifth, or fourth, follow-up visit showed improved, stabilised, or worsening illness symptom severity, as assessed by the CGI-I**.

The proportion of service users who showed improvements, stabilisation, or worsening in illness severity are visualised in Fig. 2. Service users, on average, experienced a modest improvement (M = 3·69, SD = 0·71) by their fifth (or fourth) follow-up, with most participants being assessed as having experienced ‘no change’ (i.e., stabilised; n = 451, 60·8%) or improvements (n = 243, 32·6%) in their symptom severity. For functional improvement, a significant decrease in median disability summary scores was observed at the fifth (or fourth) follow-up assessment (Median_intake_ = 62·50, Median_follow-up_ = 58·33, *z* = 6·27, *p* < 0·001), after an average of 9·9 (SD = 4·9) weeks.

#### Predictors of clinical improvement (n = 742)

All assumptions for multinomial logistic regression were tested and met. No evidence of multicollinearity amongst predictor variables was detected, with variance inflation factors (VIFs) ranging from 1·05 to 2·15. Step 1 (sociodemographic variables) was statistically significant (Model fit chi-square = 62·64, df = 16, *p* < 0·001) and accounted for 10·1% of the variance in clinical improvement (Nagelkerke *R*^*2*^ = 0·101). Step 2 (clinical variables) resulted in an increase of 1·8% in variance (Nagelkerke *R*^*2*^ = 0·119), while Step 3 (service-related variables) resulted in an increase of 9·4% in variance - explaining 21·3% of variance (Nagelkerke *R*^*2*^ = 0·213) in clinical improvement - and a statistically significant (Model fit chi-square = 128·80, df = 42, *p* < 0·001) regression model.

Using ‘worsening’ as the reference category, being female (OR = 0·48, *p* = 0·041), and living further away, between 20 and 100 km, from the CMHT office (OR = 0·32, *p* = 0·033) were associated with lessened odds of symptom stabilisation. For both sexes, receiving pharmacological intervention(s) (OR = 1·43, *p* = 0·005) significantly predicted symptom improvement, while service users receiving CMHT care in conflict-exposed oblasts had significantly lower odds of symptom improvement (OR = 0·25, *p* = 0·002).

Using ‘stabilisation’ as the reference category, being female (OR = 2·10, *p* = 0·041) and living at a distance (20–100 km) from the CMHT office (OR = 2·77, *p* = 0·033) were associated with greater odds of worsening symptoms. Reduced odds of symptom improvement, compared to stabilisation were associated with the following variables: receiving more general health services (OR = 0·80, *p* < 0·001), having a greater number of hospitalisations in the 12 months prior to intake (OR = 0·76, *p* = 0·009), more frequent use of psychosocial services for the service user themselves (OR = 0·87, *p* = 0·027) and for the service users' family (OR = 0·86, *p* = 0·027), and receiving CMHT care in conflict-exposed oblasts (OR = 0·26, *p* < 0·001). No variables significantly predicted greater odds of symptom improvement compared to stabilisation (Table 4).

**Table 4:** Results of the final multinomial logistic regression for clinical improvement for 742 CMHT service users who had a CGI-I score at fifth, or fourth, follow-up visit.

Predictor	Reference = Worsening						Reference = Stabilisation					
	Stabilisation			Improvement			Worsening			Improvement		
	OR	*p*-value	95% CI	OR	*p*-value	95% CI	OR	*p*-value	95% CI	OR	*p*-value	95% CI
**Sociodemographic variables**												
Sex (female)^c^	0·48	0·041^a^	(0·23, 0·97)	0·67	0·300	(0·32, 1·42)	2·10	0·041^a^	(1·03, 4·29)	1·41	0·070	(0·97, 2·06)
Age	1·01	0·623	(0·98, 1·04)	1·00	0·798	(0·97, 1·03)	0·99	0·623	(0·97, 1·02)	0·99	0·165	(0·97, 1·00)
Distance from CMHT office (20–100 km)	0·36	0·033^a^	(0·14, 0·92)	0·58	0·279	(0·22, 1·55)	2·77	0·033^a^	(1·08, 7·09)	1·62	0·085	(0·94, 2·79)
Time since intake	1·03	0·286	(0·98, 1·07)	1·00	0·892	(0·96, 1·05)	0·98	0·286	(0·93, 1·02)	0·98	0·156	(0·95, 1·00)
Employment status (employed)	0·45	0·226	(0·12, 1·64)	0·82	0·778	(0·21, 3·24)	2·23	0·226	(0·61, 8·13)	1·83	0·114	(0·86, 3·88)
Employment status (unemployed)	0·77	0·642	(0·26, 2·30)	1·03	0·958	(0·32, 3·30)	1·30	0·642	(0·43, 3·86)	1·34	0·345	(0·73, 2·44)
Completed tertiary education (yes)	0·81	0·592	(0·37, 1·77)	0·77	0·539	(0·33, 1·78)	1·24	0·592	(0·57, 2·72)	0·95	0·836	(0·61, 1·50)
Location (conflict-exposed)	0·96	0·928	(0·44, 2·11)	0·25	0·002^a^	(0·10, 0·61)	1·04	0·928	(0·47, 2·27)	0·26	<0·001^b^	(0·15, 0·46)
**Clinical variables**												
Presence of somatic comorbidity (yes)	0·87	0·703	(0·41, 1·82)	0·71	0·405	(0·32, 1·58)	1·16	0·703	(0·55, 2·43)	0·83	0·375	(0·54, 1·26)
No. of hospitalisations in the 12 months prior to intake	0·98	0·910	(0·73, 1·33)	0·74	0·091	(0·53, 1·05)	1·02	0·910	(0·75, 1·38)	0·76	0·009^a^	(0·61, 0·93)
ICD-10 diagnosis F20s (yes)	0·60	0·249	(0·25, 1·44)	0·43	0·070	(0·17, 1·07)	1·68	0·249	(0·70, 4·05)	0·72	0·133	(0·47, 1·11)
**Services received and obtaining ‘community outreach’ goal (≥ 50% of visits provided in service user's community)**												
Pharmacological services	1·26	0·057	(0·99, 1·61)	1·43	0·005^a^	(1·11, 1·84)	0·79	0·057	(0·62, 1·01)	1·13	0·052	(0·99, 1·28)
General health services	1·06	0·591	(0·86, 1·31)	0·85	0·161	(0·68, 1·07)	0·94	0·591	(0·76, 1·17)	0·80	<0·001^b^	(0·71, 0·90)
Psychosocial services (for service user)	0·93	0·524	(0·74, 1·17)	0·81	0·092	(0·64, 1·04)	1·08	0·524	(0·86, 1·36)	0·87	0·027^a^	(1·02, 1·29)
Psychosocial services (for service user's family)	0·95	0·686	(0·76, 1·20)	0·82	0·116	(0·64, 1·05)	1·05	0·686	(0·83, 1·32)	0·86	0·027^a^	(0·76, 0·98)
Social services	1·26	0·330	(0·79, 2·00)	1·19	0·495	(0·73, 1·94)	0·79	0·330	(0·50, 1·27)	0·94	0·617	(0·75, 1·19)
Connecting to other services	0·80	0·201	(0·58, 1·12)	0·82	0·301	(0·56, 1·19)	1·24	0·201	(0·89, 1·74)	1·02	0·877	(0·80, 1·30)
Support to integrate into the community	1·70	0·409	(0·48, 6·02)	1·83	0·358	(0·50, 6·66)	0·59	0·409	(0·17, 2·08)	1·08	0·744	(0·69, 1·67)
Developing a recovery plan	1·14	0·441	(0·82, 1·58)	1·20	0·307	(0·85, 1·69)	0·88	0·441	(0·63, 1·22)	1·05	0·558	(0·89, 1·24)
Developing a treatment plan	1·09	0·735	(0·67, 1·76)	1·19	0·491	(0·73, 1·96)	0·92	0·735	(0·57, 1·49)	1·10	0·417	(0·88, 1·36)
Receiving services in the community at ≥50% of visits (yes)	0·92	0·813	(0·44, 1·89)	1·21	0·628	(0·56, 2·60)	1·09	0·813	(0·53, 2·25)	1·32	0·183	(0·88, 1·98)

OR = odds ratio; CI = confidence interval.

a *p* < 0·05.

b *p* < 0·001, denoting statistically significant thresholds.

c Predictor category for categorical variables included in parentheses ().

#### Predictors of functional improvement (n = 304)

All assumptions underpinning a hierarchical multiple linear regression were tested and met. Multicollinearity was not indicated amongst predictor variables, with VIFs ranging from 1·05 to 2·15. Variables were entered in the same stepped order as the hierarchical multinomial regressions for clinical improvement (see above). Step 1 (sociodemographic variables) of the model was statistically significant in predicting change in disability score (*F* (8282) = 2·82, *p* = 0·005, *R*^*2*^ = 0·07). Step 2 (clinical variables) was also statistically significant (*F* (11,279) = 2·86, *p* = 0·001, *R*^*2*^ = 0·10) and resulted in a statistically significant increase in *R*^*2*^ of 0·03 (*F*_change_ (3279) = 2·85, *p* = 0·038). Step 3 (service-related variables) resulted in a significant increase in *R*^*2*^ of 0·15 (*F*_change_ (10,269) = 5·41, *p* < 0·001). This third model significantly predicted (*F* (21,269) = 4·31, *p* < 0·001) and accounted for 25% of variance (*R*^*2*^ = 0·25) in change in WHODAS scores.

Living between 20 and 100 km from the CMHT office (B = −6·95, SE = 3·38, *p* = 0·041), presenting with a somatic comorbidity (B = −5·72, SE = 2·41. *p* = 0·018), receiving more support for community integration (B = −12·93, SE = 2·79, *p* < 0·001) and more psychosocial services for the service user's family (B = −3·29, SE = 0·75, *p* < 0·001) significantly predicted improvements in disability scores. Having spent more time in CMHT care (B = 0·52, SE = 0·25, *p* = 0·039), being employed (B = 9·17, SE = 4·40, *p* = 0·038), and receiving more support in being connected to other services in the community (B = 2·88, SE = 1·31, *p* = 0·030) significantly predicted worsening disability scores (Table 5).

**Table 5:** Results of the final multiple linear regression model of functional improvement for 304 CMHT service users who had WHODAS summary scores at fifth, or fourth, follow-up visit.

Predictor	B	SE B	β	*p*-value	95% CI
**Sociodemographic variables**					
Age	−0·38	0·09	−0·03	0·657	(−0·21, 0·13)
Sex (female)^c^	0·64	2·18	0·02	0·770	(−3·65, 4·93)
Distance from CMHT office (20–100 km)	−6·95	3·38	−0·12	0·041^a^	(−13·61, −0·30)
Time since intake	0·52	0·25	0·13	0·039^a^	(0·26, 1·02)
Employment status (employed)	9·17	4·40	0·15	0·038^a^	(0·50, 17·84)
Employment status (unemployed)	1·54	3·46	0·03	0·655	(−5·26, 8·35)
Completed tertiary education (yes)	−3·24	2·58	−0·07	0·210	(−8·32, 1·84)
Location (conflict-exposed)	0·35	2·76	0·01	0·899	(−5·08, 5·78)
**Clinical variables**					
Presence of somatic comorbidity (yes)	−5·72	2·41	−0·14	0·018^a^	(−10·46, −0·97)
No. of psychiatric hospitalisations in the 12 months prior to intake	0·13	1·08	0·01	0·905	(−2·00, 2·25)
ICD-10 diagnosis F20s (yes)	−2·86	2·51	−0·07	0·255	(−7·80, 2·08)
**Services provided and obtaining ‘community outreach’ goal (≥ 50% of visits provided in service user's community)**					
Pharmacological services	0·15	0·73	0·01	0·840	(−1·28, 1·58)
General health services	0·26	0·67	0·02	0·705	(−1·07, 1·58)
Psychosocial services (for service user)	1·10	0·68	0·10	0·107	(−0·24, 2·44)
Psychosocial services (for service user's family)	−3·29	0·75	−0·26	<0·001^b^	(−4·75, −1·82)
Social services	−0·86	1·33	−0·04	0·519	(−3·48, 1·76)
Connecting to other services	2·88	1·31	0·12	0·030^a^	(0·29, 5·46)
Support to integrate into the community	−12·93	2·79	−0·26	<0·001^b^	(−18·42, −7·45)
Developing a recovery plan	1·41	1·01	0·08	0·165	(−0·58, 3·40)
Developing a treatment plan	−0·53	1·36	−0·22	0·699	(−3·21, 2·15)
Receiving services in the community at ≥50% of visits (yes)	3·16	2·35	0·08	0·179	(−1·46, 7·78)

B = unstandardised regression coefficient; SE B = standard error of unstandardised regression coefficient; β = standardised regression coefficient; CI = confidence interval.

a *p* < 0·05.

b *p* < 0·001, denoting statistically significant thresholds.

c Predictor category for categorical variables included in parentheses ().

## Discussion

The current study offers the first comprehensive, descriptive account of individuals enrolled in Ukraine's national CMHT programme. Consistent with their mandate, our findings first highlight that the Ukrainian CMHTs are enrolling people with SMI who are experiencing significant impairment.

The most common CMHT referral sources came from psychiatrists. This finding points to engagement and acceptability of the CMHT service model among this cadre, deemed necessary for the integration of the CMHT service within Ukraine's health system.^5^ This is particularly notable given the dominance of traditional clinical attitudes noted in this group,^14^ with further engagement required if Ukrainian psychiatric services are to move from “mere places of containment”, to acting as spaces that “foster the development of community-based” service provision.^15^^(p871)^ That self-referral was the second most common referral source evinces similar acceptability of the CMHT programming among service users, at least initially, and speaks to the potential of CMHTs to overcome multiple barriers that have traditionally dissuaded Ukrainians from seeking mental health support, such as distrust in the public healthcare system and mental health professionals, and low awareness of the availability of non-hospital based services for SMI.^8^ Acceptability of the CMHT programme is further evidenced by the presence of both male and female CMHT service users in a context where men and women endorse the need for mental health support at comparable rates.^16^ This acceptability aligns with previous research on CMHTs in England,^17^^,^^18^ Australia, and Canada,^17^ as well as more recent evidence from recovery-oriented community mental health services in Italy.^19^

The most frequently administered service was ‘psychosocial services (for service user)’, followed by ‘pharmacological services’, administered at slightly less than half the rate. This ratio is promising, as prioritising psychosocial over pharmacological services is one of the key indicators of CMHT programming offering recovery-oriented care (Table 1). It also signals a shift towards a stronger biopsychosocial model of service provision, which is a key goal of Ukraine's ongoing mental healthcare reform.^1^ Furthermore, the frequent provision of psychosocial services suggests these supports are being adequately planned and financed within the CMHT programming, sidestepping a common barrier seen in other Ukrainian mental health service frameworks.^15^ However, ‘psychosocial services’ is a broad term that still lacks specificity, and we were unable to differentiate between the types of psychosocial services provided. Killaspy and colleagues refer to this lack of clarity as a “fundamental issue”^20^^(p97)^ in community-based mental health programming. Similarly, Suvalo & Borovets^15^ posit that an insufficient classification of psychosocial services serves as a major hindrance to Ukraine's ongoing reform efforts, limiting insights into which psychosocial interventions contribute most to service user recovery.

Our findings further suggest that services focused on integrating service users into the community were infrequent. Despite this, we found the strongest association between support for community integration and improvements in service user functioning. While previous evaluative CMHT research from England suggests this is not always the case,^21^ our finding is consistent with various conceptual frameworks, which highlight re-integration into community life as a core component of recovery for people living with SMI.^22^^,^^23^

In relation to *where* CMHT services were delivered, slightly more than 50% of CMHT visits recorded in this study were conducted as community outreach, reflecting achievement of another key indicator of CMHT programming success (Table 1). This is laudable, evidencing that specialised mental health workers, if trained in recovery-oriented care, are willing to move away from a historic focus on institution or office-based service provision^1^—often inaccessible for individuals with SMI^24^—towards more community-oriented practice.

Promisingly, illness symptom severity was reported to have stabilised or improved for most CMHT service users by their fifth (or fourth) follow-up visit. Likewise, significant improvements in disability scores were also reported after the fifth (or fourth) follow-up visit. Taken together, and with stabilisation widely regarded as the foundation to mental health recovery,^25^ these represent early encouraging findings for Ukraine's CMHT programme. However, given wide endorsement of the multi-faceted nature of recovery for people living with SMI, with clinical recovery as only one component,^23^ additional research is required to examine possible concurrences between the programme's more traditional, clinical measures of recovery and people's subjective aspects of recovery, such as living an independent and satisfying life alongside the possible limitations of one's symptoms.^22^ A useful model to guide this research is the CHIME framework, which conceptualises personal recovery across five recovery processes—connectedness, hope and optimism about the future, identity, meaning in life, and empowerment—providing subordinate processes for each that could be used to inform outcome indicators.^23^ Moreover, as previous research indicates, the potential for incongruence between clinician and service user perspectives on clinical improvement, specifically using the CGI-I,^26^ suggests that CMHTs may benefit from the addition of more service user-centric assessments, such as the Patient Global Impression of Improvement Scale, which is derived from, and demonstrates strong concurrent validity, with the CGI.^27^

Results from regression analyses suggest that, while having no predictive effect on symptom improvement, being female was associated with increased odds of symptoms worsening compared to stabilisation. That females had slightly less than half the odds of achieving symptom stabilisation compared to males indicates that the CMHT programming falls short of providing equally effective treatment to nearly half of its service users. One potential explanation is that Ukrainian women, as traditional primary caregivers, likely have additional stressors and/or less capacity to fully engage with CMHT services.^16^ This gendered role may in turn hinder recovery; highlighting the need to bridge this gender gap in CMHT effectiveness. Doing so may necessitate consideration of adaptations to CMHT service delivery or staff training/supervision to better meet the needs of female service users. The WHO's manual for gender responsive health services may be a useful resource in this endeavour.^28^

Conflicting results on CMHT service proximity in this study—in terms of predicting both symptom improvement and greater functional impairment—are inconsistent with previous research on the importance of geographically accessible mobile health support in Ukraine,^29^ and community-mental health support in other contexts.^30^ Service users who were employed also had reduced odds of exhibiting functional improvement, which contradicts previous longitudinal research demonstrating no association between employment and functional outcomes among individuals with SMI.^31^ Longer-term research is recommended to better elucidate the influence of both variables on clinical recovery.

That receiving CMHT care within a conflict-exposed oblast (Donetska) was associated with reduced odds of clinical improvement reflects the well-documented impact of conflict-exposure on the onset and exacerbation of mental disorders.^32^ There is also the well-established challenge of providing effective community mental health and psychosocial support (MHPSS) in conflict settings, which has already been documented specifically in Ukraine's Donetska oblast.^33^ This finding supports *The Commission's* call for additional training for CMHT staff being deployed in conflict-exposed oblasts (which has increased since the escalation of the war in 2022), particularly regarding trauma-informed approaches to service provision.^1^

The potential for geographic expansion, as well as enhanced capacity-building for CMHTs operating in conflict-exposed oblasts, will, however, require addressing the shortage of qualified mental health workers—a cadre that has been steadily diminishing since the escalation of the war, particularly in conflict-exposed regions.^34^ Ensuring sufficient CMHT staffing may, at least in the short term, depend on maintaining international aid for MHPSS,^35^ a significant challenge considering recent drops in funding to Ukraine.^36^

Recent research conducted among mental health practitioners in Ukraine also calls for the implementation of evidence-based strategies to improve their well-being.^37^ Indeed, providing all mental health practitioners with supportive supervision is mandated by Ukrainian law.^38^ The ‘Integrated Model for Supervision’^39^—as a resource currently being integrated into the supervision practices of the country's health, social protection, and education workforces^40^—may be useful in this regard. Taken together, ensuring that CMHT staff are both of an adequate number *and* well-supported are critical to safeguard both mental health and the broader health infrastructure amid the current war.^1^

Previous research shows that people with greater disability or complex medical needs often face additional challenges to functional recovery.^41^ Our findings suggest this relationship may be more nuanced in the Ukrainian context. A greater number of hospitalisations prior to CMHT intake and receiving more general health services from the CMHT were associated with reduced odds for improvement in symptom severity. Likewise, being connected to other community services and having spent more time in CMHT care predicted worsening functional impairment. Conversely, service users with a somatic comorbidity demonstrated greater odds for functional improvement. This discrepancy may be partly explained by individuals with both a mental and somatic disorder experiencing higher baseline functional impairment,^42^ thus creating more room for improvement from CMHT services. As such, the CMHTs' engagement with primary healthcare centres for the collaborative management of somatic comorbidities in Ukrainians with SMI^3^ requires further investigation.

Pharmacological intervention being associated with clinical improvement in comparison to symptom worsening is consistent with a well-established literature suggesting that people living with SMI respond well to some level of pharmacological assistance to alleviate symptomology^43^ and supports its common use by the Ukrainian CMHTs (Table 3). However, its lack of association with *functional improvement*, as gleaned from responses provided by the service user, raises questions about the possibility of clinician bias influencing its predictive effect. Our findings suggest that functional impairment was more influenced by the provision of psychosocial services (for the service user's family) and support for community integration. The former highlights the importance of fostering a well-supported familial network in recovery, particularly given families' substantial caregiving role.^44^ Alongside pharmacotherapy's role in clinical improvement, the positive impact of familial and community support ultimately underscores the need for a biopsychosocial model of mental healthcare, where both pharmacotherapy and services that address the psychosocial drivers of functional and clinical impairment (such as the influence of an individual's external environment) are central to recovery.^45^

The current study is not without limitations. First, its observational nature limits our ability to attribute observed changes in outcomes to Ukraine's CMHT programme specifically. Further, absence of a comparison group limits our ability to speak to the advantages of Ukraine's CMHT programme over other models of care.

The use of clinical and functional outcome measures in this study may emphasise the importance of symptom alleviation over other, more personal measures of recovery. Similarly, use of the CGI-I to assess symptom severity *compared* to intake score may be prone to recall bias^26^ and/or poor interrater reliability in instances where follow-up assessment was conducted by a different member of the CHMT. That clinical improvement was assessed by a member of the CMHT also meant potential over-estimation of the effect of those services as a form of indirect self-assessment.^46^ This may be particularly the case in the Ukrainian context, given the historic predominance of pharmacotherapy and specialist-led mental health services.^14^

The order of entry used in hierarchical regression techniques posed another limitation, since they are prone to subjectivity bias. Additionally, we did not adjust for potential clustering of participants within the 12 oblasts, which may have led to underestimated standard errors and inflated significance levels. Moreover, beyond pairwise deletion, we did not apply any methods to account for missing data within our regression analyses, most of which pertained to the WHODAS outcome measure. This may introduce additional complications in interpreting our results related to functional improvement.

Lastly, given that our study includes analysis of service user data from the ‘service establishment’ phase only, the long-term outcomes of service users, including any differences in recovery progression across CMHT service users with different clinical profiles, remains unknown. Overall, these limitations restrict our ability to generalise our findings to other Ukrainians with SMI, despite the results presented here still offering important insights to guide future CMHT programming in Ukraine.

Future evaluations of Ukraine's CMHT programme would benefit from documenting specific type(s) of psychosocial services delivered at each visit, incorporating personal recovery measures (including qualitative enquiries), and including community integration and service user collaboration indicators into revised CMHT standard operating procedures. Service user collaboration is considered particularly relevant given the importance of autonomy and agency in the recovery process,^22^ and, as Menear posits,^14^ that treating services users as “equal partners” in the “planning, provision, and evaluation of mental health services” will be key to Ukraine's ongoing reform efforts.^(p869)^ Documenting this process presents an opportunity to assess the extent to which the CMHT programme is person-centred in its application,^3^ as well as to further learnings, and maximise recovery outcomes—including those of a non-clinical nature. Finally, longer observational periods are required to capture the complex nature of recovery of service users enrolled in Ukraine's CMHT programme. Conducting such research will also provide a crucial opportunity to re-examine our findings in light of the current war, which has likely had a deleterious effect on CMHT service provision in Ukraine.

In conclusion, we found promising results associated with the establishment phase of Ukraine's CMHT programme. Most people receiving services from a CMHT, for a minimum of five follow-up visits, demonstrated clinical and functional signs of stabilisation or improvement. Moreover, CMHTs were successful in achieving key performance indicators in relation to what services were delivered and where they were delivered. Additional resources to address noted discrepancies on clinical improvement among female service users and those located in conflict-affected areas are warranted. Likewise, the discrepancies between influence of pharmacological and psychosocial services (for the service user's family) and community integration on service user *functional improvement* may reflect an over-reliance on symptomology as a marker of recovery and reinforces the need to consider a mix of personal and clinical measures of mental health and wellbeing. Taken together, our findings support the Ukrainian CMHT model as a promising mode of mental health service delivery. Further research to assess Ukraine's CMHTs' long-term impacts and efficacy, compared with other models of mental health care, will secure additional evidence for this approach and strengthen the rationale for its continued implementation.

## Contributors

**AL:** Study conceptualisation, study design, data interpretation, manuscript writing, project administration, oversight, funding acquisition.

**CZ:** Study conceptualisation, study design, verification of underlying data, design of tables/figures, data preparation, data analysis, data interpretation, manuscript writing, project administration.

**MRB:** Study conceptualisation, study design, design of tables/figures, data analysis, data interpretation, manuscript writing.

**OK:** Verification of underlying data, design of tables/figures, data preparation.

**AS:** Study conceptualisation, data interpretation, manuscript writing, oversight, funding acquisition.

**BA:** Study conceptualisation, study design, oversight, funding acquisition.

**DC:** Study conceptualisation, oversight.

**PH:** Data analysis, data interpretation.

**MS:** Data preparation.

**KD:** Data preparation.

**IM:** Data preparation, oversight.

**JH:** Oversight.

**FV:** Study conceptualisation, study design, design of tables/figures, data analysis, data interpretation, oversight, project administration.

All authors have seen and approved the initial version of this manuscript, and the final version was seen and approved by AL, CZ, AS, and FV.

## Data sharing statement

Data is accessible via contact with the WHO Country Office in Ukraine and given reasonable request.

## Editor note

The Lancet Group takes a neutral position with respect to territorial claims in published maps and institutional affiliations.

## Declaration of generative AI and AI-assisted technologies in the writing process

During the preparation of this work the author(s) used ChatGPT-4 in order to improve the readability and language of the manuscript. After using this tool/service, the author(s) reviewed and edited the content as needed and take(s) full responsibility for the content of the published article.

## Declaration of interests

AL, DC, AS, OK, JH are currently employed by the WHO. BA was previously employed by the WHO and IM was previously employed by the Ministry of Health of Ukraine as a Deputy Minister of Health. These authors supported initial development and rollout of the Ukrainian CMHT programming. All other authors declare no conflicts of interests.

## Appendix A Supplementary data

Supplementary Tables S1–S3

Translated Abstract-Ukrainian
