# Supplementary material for: Clinical and functioning outcomes during the establishment phase of Ukraine's community mental health teams: a descriptive analysis
Source: Lancet Reg Health Eur. 2025 Sep 3;58:101446. doi: 10.1016/j.lanepe.2025.101446 (PMC12446188; doi:10.1016/j.lanepe.2025.101446)
Supplement: Supplementary Tables S1–S3 [file mmc1.docx]

***Supplementary Table 1:* Number of CMHT service users at CMHT intake and the first 10 follow-up visits**

| **CMHT visit** | **Number of service users with available data** |
| --- | --- |
| Intake | 945 |
| Follow-up visit 1 | 905 |
| Follow-up visit 2 | 863 |
| Follow-up visit 3 | 821 |
| Follow-up visit 4 | 780 |
| Follow-up visit 5 | 748 |
| Follow-up visit 6 | 704 |
| Follow-up visit 7 | 647 |
| Follow-up visit 8 | 585 |
| Follow-up visit 9 | 526 |
| Follow-up visit 10 | 470 |

***Supplementary Table 2:* Differences across CMHT service user demographic and clinical variables between service users with at least five follow-up visits (n = 748) and those with less than five follow-up visits (n = 199)**

| **Variable** |  | **Service users with at least five follow-up visits (n = 748)** | **Service users with less than five follow-up visits (n = 199)** | **Test Statistic(df)** | ***p*-value** |
| --- | --- | --- | --- | --- | --- |
|  |  |  |  |  |  |
|  |  | n (%) | n (%) |  |  |
| Sex |  |  |  |  |  |
|  | Male | 399 (53·6%) | 105 (52·8%) | χ^2^(1) = 0·040 | 0·842 |
|  | Female | 425 (46·4%) | 94 (47·2%) |  |  |
| Distance from the CMHT office (in km) |  |  |  | χ^2^(4) = 2·36 | 0·669 |
|  | <5km | 224 (30·3%) | 55 (28·4%) |  |  |
|  | <20km | 409 (55·3%) | 112 (57·7%) |  |  |
|  | <50km | 73 (9·9%) | 22 (11·3%) |  |  |
|  | <100km | 30 (4·1%) | 4 (2·1%) |  |  |
| Education level |  |  |  | *Fisher’s exact test* = 11·71 | 0·033* |
|  | Primary of incomplete secondary education | 43 (5·8%) | 4 (2·1%) |  |  |
|  | Basic secondary education | 145 (19·6%) | 37 (19·2%) |  |  |
|  | Vocational-technical secondary education | 342 (46·2%) | 90 (46·6%) |  |  |
|  | Incomplete tertiary education | 55 (7·4%) | 9 (4·7%) |  |  |
|  | Tertiary education | 155 (20·9%) | 52 (26·9%) |  |  |
| Employment status |  |  |  | χ^2^(5) = 24·38 | < 0·001** |
|  | Employed (full-time) | 30 (4·1%) | 20 (10·4%) |  |  |
|  | Employed (part-time) | 38 (5·1%) | 20 (10·4%) |  |  |
|  | Self-employed | 18 (2·4%) | 2 (1·0%) |  |  |
|  | Unemployed (but registered at employment centre) | 5 (0·7%) | 2 (1·0%) |  |  |
|  | Unemployed (and not registered at the employment centre) | 556 (75·1%) | 119 (62·0%) |  |  |
|  | Other occupation*** | 93 (12·6%) | 29 (15·1%) |  |  |
| Location |  |  |  | χ^2^(13) = 120·48 | < 0·001** |
|  | Zhytomyrska | 32 (4·3%) | 22 (11·1%) |  |  |
|  | Khersonska | 62 (8·3%) | 34 (17·1%) |  |  |
|  | Zakarpatska | 31 (4·1%) | 12 (6·0%) |  |  |
|  | Chernivetska | 77 (10·3%) | 12 (6·0%) |  |  |
|  | Kyivska | 46 (6·1%) | 2 (1·0%) |  |  |
|  | Khmelnytska | 66 (8·8%) | 6 (3·0%) |  |  |
|  | Donetska | 171 (22·8%) | 63 (31·6%) |  |  |
|  | Mykolaivska | 65 (8·7%) | 11 (5·5%) |  |  |
|  | Cherkaska | 28 (3·7%) | 14 (7·0%) |  |  |
|  | Volynska | 62 (8·3%) | 10 (5·0%) |  |  |
|  | Chernihivska | 66 (8·8%) | 9 (4·5%) |  |  |
|  | Ivano-Frankivska | 42 (5·6%) | 4 (2·0%) |  |  |
| Referral source* |  |  |  | *Fisher’s exact test* = 23·42 | < 0·001** |
|  | Outpatient psychiatrist | 330 (44·5%) | 111 (56·3%) |  |  |
|  | Self-referral | 197 (26·6%) | 35 (17·8%) |  |  |
|  | Inpatient psychiatrist | 143 (19·3%) | 35 (17·8%) |  |  |
|  | Family doctor | 22 (3·0%) | 13 (6·6%) |  |  |
|  | Social service | 1 (0·1%) | 0 (0·0%) |  |  |
|  | Other | 48 (6·5%) | 3 (1·5%) |  |  |
| ICD-10 diagnosis |  |  |  | χ^2^(3) = 13·61 | 0·003* |
|  | Schizophrenia, schizotypal and delusion disorders (F20-29) | 541 (73·5%) | 128 (67·7%) |  |  |
|  | Mood [affective] disorders (F30-39) | 85 (11·5%) | 24 (12·7%) |  |  |
|  | Anxiety, dissociative, stress-related, somatoform, and other non-psychotic disorders (F40-48) | 27 (3·7%) | 19 (10·1%) |  |  |
|  | Other (F0-09; 50-59; 60-69 & 70-79) | 83 (11·3%) | 18 (9·5%) |  |  |
| Presence of somatic comorbidity |  |  |  | χ^2^(1) = 8·11 | 0·004* |
|  | Yes | 259 (34·8%) | 48 (24·1%) |  |  |
|  | No | 486 (65·2%) | 151 (75·9%) |  |  |
|  |  | M (SD) | M (SD) |  |  |
| Age |  | 44·51 (13·97) | 43·06 (14·41) | *F* (1,933) = 0·62 | 0·200 |
| CGI intake score (CGI-S) |  | 5·03 (1.00) | 4·89 (1·01) | *F (*1,934) = 0·39 | 0·077 |
|  |  |  |  |  |  |
| Number of psychiatric hospitalisations in the 12 months before intake* |  | 0·94 (1·02) | 0·69 (0·80) | *F* (1,928) = 2·26 | 0·002* |
| WHODAS summary score at intake |  | 62·07 (18·04) | 57·47 (21·13) | *F* (1,886) = 9·17 | 0·003* |

Note: n and % do not include missing data, where applicable

*p < 0·05 and ******p < 0·001, denoting statistically significant thresholds

***Other occupation categories included: manages household, pensioner and manages household, on parental leave, studying, pensioner

***Supplementary Table 3*: Differences across CMHT service user demographic and clinical variables between service users included in the functional improvement analysis (n = 304) and those excluded from the functional improvement analysis (n = 444)**

| **Variable** |  | **Service users eligible for the functional improvement analysis (n = 304)** | **Service users ineligible for the functional improvement analysis (n = 444)** | **Test Statistic(df)** | ***p-*value** |
| --- | --- | --- | --- | --- | --- |
|  |  |  |  |  |  |
|  |  | n (%) | n (%) |  |  |
| Sex |  |  |  |  |  |
|  | Male | 161 (53·1%) | 238 (53·8%) | χ^2^(1) = 0·04 | 0·848 |
|  | Female | 142 (46·9%) | 204 (46·2%) |  |  |
| Distance from the CMHT office (in km) |  |  |  | χ^2^(4) = 20·78 | < 0·001** |
|  | <5km | 104 (34·3%) | 120 (27·5%) |  |  |
|  | <20km | 173 (57·1%) | 236 (54·0%) |  |  |
|  | <50km | 23 (7·6%) | 50 (11·4%) |  |  |
|  | <100km | 2 (0·7%) | 28 (6·4%) |  |  |
| Education |  |  |  | χ^2^(4) = 6·39 | 0·172 |
|  | Primary or incomplete secondary education | 22 (7·3%) | 21 (4·8%) |  |  |
|  | Basic secondary education | 67 (22·1%) | 78 (17·8%) |  |  |
|  | Vocational-technical secondary education | 140 (46·2%) | 202 (46·2%) |  |  |
|  | Incomplete tertiary education | 20 (6·6%) | 35 (8·0%) |  |  |
|  | Tertiary education | 54 (17·8%) | 101 (23·1%) |  |  |
| Employment status |  |  |  | χ^2^(5) = 32·54 | < 0·001** |
|  | Employed (full-time) | 12 (4·0%) | 18 (4·1%) |  |  |
|  | Employed (part-time) | 24 (8·0%) | 14 (3·2%) |  |  |
|  | Self-employed | 9 (3·0%) | 9 (2·1%) |  |  |
|  | Unemployed (but registered at employment centre) | 3 (1·0%) | 2 (0·5%) |  |  |
|  | Unemployed (and not registered at the employment centre) | 196 (65·1%) | 360 (82.0%) |  |  |
|  | Other occupation*** | 57 (18·9%) | 36 (8·2%) |  |  |
| Location |  |  |  | χ^2^(13) = 223·05 | < 0·001** |
|  | Zhytomyrska | 25 (8·2%) | 7 (1·6%) |  |  |
|  | Khersonska | 41 (13·5%) | 21 (4·7%) |  |  |
|  | Zakarpatska | 24 (7·9%) | 7 (1·6%) |  |  |
|  | Chernivetska | 53 (17·4%) | 24 (5·4%) |  |  |
|  | Kyivska | 19 (6·3%) | 27 (6·1%) |  |  |
|  | Khmelnytska | 7 (2·3%) | 59 (13·3%) |  |  |
|  | Donetska | 17 (5·6%) | 154 (34·6%) |  |  |
|  | Mykolaivska | 51 (16·8%) | 14 (3·2%) |  |  |
|  | Cherkaska | 6 (2·0%) | 22 (5·0%) |  |  |
|  | Volynska | 26 (8·6%) | 36 (8·1%) |  |  |
|  | Chernihivska | 17 (5·6%) | 49 (11·0%) |  |  |
|  | Ivano-Frankivska | 18 (5·9%) | 24 (5·4%) |  |  |
| Referral source |  |  |  | χ^2^(5) = 34·01 | < 0·001** |
|  | Outpatient psychiatrist | 104 (34·3%) | 226 (51·6%) |  |  |
|  | Self-referral | 90 (29·7%) | 107 (24·4%) |  |  |
|  | Inpatient psychiatrist | 64 (21·1%) | 79 (18·0%) |  |  |
|  | Family doctor | 11 (3·6%) | 11 (2·5%) |  |  |
|  | Social service | 0 (0·0%) | 1 (0·2%) |  |  |
|  | Other | 34 (11·2%) | 14 (3·2%) |  |  |
| ICD-10 diagnosis |  |  |  | χ^2^(3) = 12·78 | 0·005* |
|  | Schizophrenia, schizotypal and delusion disorders (F20-29) | 212 (70·9%) | 329 (75·3%) |  |  |
|  | Mood [affective] disorders (F30-39) | 28 (9·4%) | 57 (13·0%) |  |  |
|  | Anxiety, dissociative, stress-related, somatoform, and other non-psychotic disorders (F40-48) | 18 (6·0%) | 9 (2·1%) |  |  |
|  | Other (F0-09; 50-59; 60-69 & 70-79) | 41 (13·7%) | 42 (9·6%) |  |  |
| Presence of somatic comorbidity |  |  |  | χ^2^(1) = 0·27 | 0·601 |
|  | Yes | 102 (33·7%) | 157 (35·5%) |  |  |
|  | No | 201 (66·3%) | 285 (64·5%) |  |  |
|  |  | M (SD) | M (SD) |  |  |
| Age |  | 46·10 (14.93) | 43·42 (13.16) | *t* (739) = -2·58 | 0·010* |
| CGI intake score (CGI-S) |  | 5·06 (0·85) | 5·01 (1·09) | *t* (740) *=* -0·67 | 0·501 |
| Number of psychiatric hospitalisations in the 12 months before intake |  | 0·82 (1·10) | 1·03 (0·96) | *F* (1,739) = 7·51 | 0·006* |
| WHODAS summary score at intake |  | 63·97 (18·60) | 60·65 (17·50) | *F* (1,707) = 5·92 | 0·015* |

Note: n and % do not include missing data, where applicable

*p < 0·05 and ******p < 0·001, denoting statistically significant thresholds

***Other occupation categories included: manages household, pensioner and manages household, on parental leave, studying, pensioner
