# Supplementary material for: Clinical and functioning outcomes during the establishment phase of Ukraine's community mental health teams: a descriptive analysis
Source: Lancet Reg Health Eur. 2025 Sep 3;58:101446. doi: 10.1016/j.lanepe.2025.101446 (PMC12446188; doi:10.1016/j.lanepe.2025.101446)
Supplement: Translated Abstract-Ukrainian [file mmc2.docx]

**Резюме**

**Передумови:** Загальнонаціональна програма Мобільних мультидисциплінарних команд з психічного здоров’я (ММК) є ключовим елементом поточної реформи психіатричної допомоги в Україні. Однак наразі не існує досліджень, які б повідомляли про вплив ММК на клінічне відновлення отримувачів послуг. Це дослідження має дві мети: (i) описати, кого залучають українські ММК, які послуги вони надають найчастіше та де саме вони надаються, а також (ii) визначити, чи спостерігаються клінічні та/або функціональні покращення серед отримувачів послуг після шести візитів до ММК (первинний + п’ять повторних), і якщо так, то виявити основні предиктори таких покращень.

**Методи:** Дані 947 отримувачів послуг ММК, залучених у період з квітня по грудень 2021 року, були проаналізовані за клінічними результатами за допомогою Шкали загального клінічного враження (CGI) та за функціональними результатами за допомогою Шкали оцінки обмеження життєдіяльності ВООЗ (WHODAS 2.0). Для оцінки змін у показниках CGI та WHODAS під час п’ятого (або четвертого) повторного візиту до ММК застосовували критерій χ² та критерій знакових рангів Вілкоксона. Ієрархічна багатономінальна логістична регресія та ієрархічна множинна лінійна регресія використовувались для виявлення предикторів клінічного та функціонального покращення відповідно.

**Результати:** Більшість отримувачів послуг були чоловіками, безробітними та мали діагноз із шизофренічного спектру. Серед отримувачів послуг, для яких були доступні дані як під час первинного, так і під час п’ятого (або четвертого) повторного візиту, зафіксовано значне покращення функціонування (Медіана_первинний візит_ = 62·50, Медіана_повторний візит_ = 58·33, *z* = -6·27, *p* < 0·001), а у більшості отримувачів послуг рівень важкості захворювання стабілізувався (n = 451/742, 60·8%) або покращився (n = 243/742, 32·6%). Клінічна стабілізація (у порівнянні з погіршенням) асоціювалася з чоловічою статтю та проживанням на відстані менш ніж 20 км від офісу ММК, тоді як клінічне покращення асоціювалося з частішим отриманням фармакологічної підтримки та наданням послуг ММК у регіонах, не охоплених бойовими діями. Функціональне покращення передбачалося проживанням на відстані 20–100 км від офісу ММК, наявністю соматичної коморбідності, частішим отриманням психосоціальної підтримки для родини отримувача, а також більшою підтримкою інтеграції у громаду.

**Інтерпретація:** Ми виявили позитивні результати, пов’язані із залученням до програми ММК в Україні. У дослідженні також надаються рекомендації для подальших досліджень і можливі шляхи вдосконалення програми ММК.

**Фінансування:** Дослідження було профінансовано в рамках Спеціальної ініціативи ВООЗ у сфері психічного здоров’я.
